# Shear rate specific blood viscosity and shear stress of carotid artery duplex ultrasonography in patients with lacunar infarction

**DOI:** 10.1186/1471-2377-13-36

**Published:** 2013-04-18

**Authors:** Seul-Ki Jeong, Robert S Rosenson

**Affiliations:** 1 Department of Neurology & Research Institute of Clinical Medicine, Chonbuk National University - Biomedical Research Institute of Chonbuk National University Hospital, San 2-20, Geumam-dong, Deokjin-gu, Jeonju, Jeonbuk, 561-180, South Korea; 2 Mount Sinai Heart, Mount Sinai School of Medicine, New York, NY, USA

**Keywords:** Vascular shear stress, Blood viscosity, Shear rate, Peak-systolic, End-diastolic

## Abstract

**Background:**

This study describes a new method for determining site-specific vascular shear stress using dynamic measures of shear rate and blood viscosity (BV) in the carotid arteries, and examines characteristics of carotid arterial shear stress among patients with lacunar infarction.

**Methods:**

Vascular shear stress measurements were conducted in 37 patients (17 lacunar infarction patients and 20 control subjects) using duplex ultrasonography. Vessel wall diameters and velocities were measured in each arterial segment at peak-systolic (PS) and end-diastolic (ED) phases, for calculation of PS/ED shear rates. PS/ED shear stresses [dyne/cm^2^] were determined with PS/ED shear rates and shear-rate dependent BV values. For comparison, both values of hematocrit-derived BV and BV measurements at 300 s^-1^ were used for calculation of shear stress.

**Results:**

All cardiovascular disease (CVD) risk factors including BV values were similar between the two groups. In both common carotid arteries, PS and ED shear stresses were significantly lower in the patients with lacunar infarction than in controls in multivariate models that included age, sex, and other major CVD risk factors. PS and ED shear stresses using the shear rate specific BV were 4.5% lower and 7.3% higher than those using the two other BVs, respectively.

**Conclusion:**

Lacunar infarction was associated with reduced carotid arterial shear stress. The use of estimated BV for calculating carotid arterial shear stress provides more accurate assessment of the hemodynamic contribution of shear stress than previous models that have arbitrarily assigned a constant value to this dynamic flow property.

## Background

The vascular system can generally be viewed as a closed system, constraining a series of biochemical processes and mechanical stresses that are regulated by various homeostatic mechanisms [1]. From this theoretical perspective, if mechanical stresses such as wall shear stresses and tensile stresses are not properly dispersed, then an artery may sustain injury. This mechanical injury ultimately results in atherothromboembolic disease and its clinical manifestations of stroke, myocardial infarction and claudication [2].

Blood is a fluid suspension of plasma and cells such as erythrocytes, leukocytes, and platelets, and it demonstrates non-Newtonian fluid mechanics. A non-Newtonian fluid has a variable, non-linear relation between blood viscosity and blood flow [3]. Specifically, blood viscosity is higher at low shear rates ($\dot{\gamma }$) and is reduced as shear rate increases [4]. This shear rate dependent aspect of blood viscosity (BV, *μ*) has presented challenges to the accurate calculation of vascular wall shear stress, the frictional force per unit area acting tangentially to the arterial wall. To date, nearly all previous studies on arterial wall shear stress have neglected the flow-dependency of blood [5] and improperly presented BV as a constant Newtonian fluid [6-8]. Although the non-Newtonian characteristics of blood have been assumed in some previous studies, BV over ranges of shear rates have not been measured directly, but the laws of viscosity were applied empirically [9,10].

This study was designed to evaluate a new method for calculating vascular shear stresses along carotid arterial segments using duplex ultrasonography and dynamic BV measurements, and to examine whether the vascular shear stress of carotid artery was different between patients with lacunar infarction and control subjects using the new method (shear rate-specific BV and shear stress). This hypothesis is based on the observation that lacunar infarction is accompanied by endothelial dysfunction [11] and low cerebral arterial blood flow velocities [12]. Finally, we compared the shear stresses of the new method with those of the conventional methods using other BV determinations such as hematocrit-derived BV or constant values at shear rate of 300 s^-1^.

## Methods

### Subjects

Seventeen patients with lacunar infarction and 20 control subjects were examined for carotid duplex ultrasonography and measured for BV at the Department of Neurology, Chonbuk National University Hospital, Jeonju, South Korea. A diagnosis of lacunar infarction was made based upon acute onset of focal neurological deficit and a relevant lesion was seen on diffusion-weighted magnetic resonance imaging (MRI) of the brain [13]. A lacunar infarct was diagnosed when the brain MRI identified a lesion less than 15 mm in diameter with characteristics of high signal intensity on diffusion-weighted imaging and low signal intensity on the apparent diffusion coefficient map. The location of the lacunar infarction included either the basal ganglia, corona radiata, thalamus, or brainstem, but not cortex [12]. The control group was defined as patients with cardiovascular risk factors but no previous history of ischemic vascular diseases (e.g. stroke, myocardial ischemia, and lower extremity arterial disease). The institutional ethics committee at Chonbuk National University Hospital approved the present study. All subjects provided written informed consent.

### Assessments and measurements

The medical history included data on prior CVD, type 2 diabetes mellitus, hypertension, dyslipidemia, and medication usage. Smoking status was recorded in pack-years. The patients were classified as hypertensive based on a persistent elevation of blood pressure (≥140/90 mmHg) or treatment with antihypertensive medications. Type 2 diabetes mellitus was defined by fasting blood glucose >7.0 mmol/L or previous use of glucose-lowering medications. Hypercholesterolemia was defined by a total cholesterol >6.2 mmol/L or low-density lipoprotein cholesterol >4.1 mmol/L, or current use of cholesterol-lowering medication. Plasma concentration of total homocysteine (tHcy) was measured by fluorescence polarization immunoassay (AxSYM, Abbott Laboratories, Abbott Park, IL).

### Carotid ultrasonography

**Figure 1:**
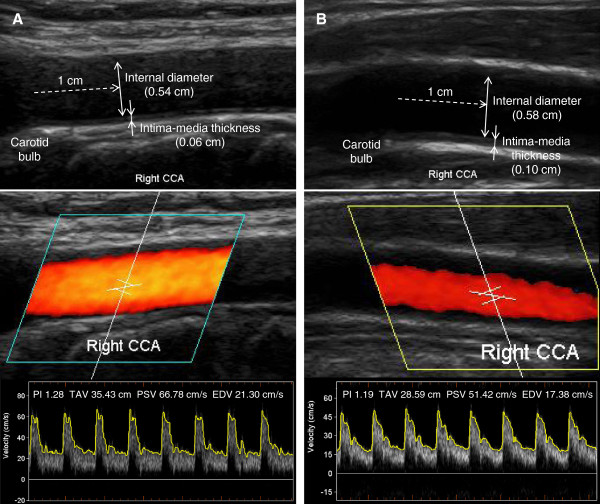
**Representative ultrasonographic measurements of right common carotid internal diameters, intima-media thicknesses (IMTs), and blood flow velocities in a control subject (A) and a patient with lacunar infarction (B).** Peak-systolic and end-diastolic internal diameters were measured at the segment 1 cm proximal to the beginning portion of carotid bulb, while IMT was measured at the peak of the R wave (end-diastole).

In the present study, internal diameter, maximum centerline velocity, and intima-media thickness (IMT) were measured bilaterally along the common carotid artery (CCA), carotid bulb, and internal carotid artery (ICA), as shown in Figure 1. To calculate site-specific shear rates, diameters and velocities of both CCAs were used [14]. In the patients with lacunar infarction, carotid ultrasonography was performed at least one week after the vascular attack. A higher-frequency 5-to-12 MHz (12 L5) linear transducer (Terason t3000, Teratech, Inc, Burlington, MA) was used with ECG triggering, and all the examinations were performed by a certified neurosonologist (SKJ) who has performed more than 5,000 cases (10,000 carotid arteries) during the past 8 years. Intra-luminal diameters were measured separately at peak-systole (PS) and end-diastole (ED) using two-dimensionally guided continuous M-mode tracings of the intimal-luminal interface of the near and far walls of carotid arterial segments, as reported previously [14]. In all segments, video images of the interfaces between lumen and intima over 5 cardiac cycles were captured, stored, and the diameters were measured from fixed images. The axial resolution of the M-mode system was 0.1 mm.

Velocities were measured at both PS and ED with the sample volume reduced to the smallest possible size (1 mm) and placed in the center of flow, thereby enabling the maximum centerline velocity to be determined. The Doppler angle was generally maintained between 45 and 55 degrees. The sample volume box was placed in the mid-lumen parallel to the vessel wall in all of the studies, as recommended [15].

The degree of ICA stenosis at both gray-scale and Doppler ultrasonography was stratified into the following 6 categories: (1) normal (no stenosis), (2) less than 50% stenosis, (3) 50~69% stenosis, (4) ≥70% stenosis to near occlusion, (5) near occlusion, and (6) total occlusion, as recommended [16].

### Exclusion criteria

The patients with the following characteristics were excluded: 1) patients who showed ICA stenosis more than 50% in at least one side, 2) patients who have been performed for carotid artery revascularization including carotid endarterectomy or stenting. Finally, patients with lacunar infarction and control subjects, who had ICA of normal or less than 50% stenosis, were included in the present study.

### Shear rate

According to the PS/ED velocities and diameters along the carotid arteries, PS/ED shear rates were determined as follows [5]:

$$\dot{\gamma }=4\times \frac{V}{D}$$

where V and D are the maximum centerline velocity and local lumen diameter, respectively. The PS and ED shear rates were calculated with corresponding PS/ED velocities and diameters, which were designated as PS shear rate ($\dot{\gamma }$_PS_) and ED shear rate ($\dot{\gamma }$_ED_).

### Blood viscosity

For a measurement of BV, blood was sampled at antecubital vein and stored in ethylenediaminetetraacetic acid (EDTA) tube. BV was measured using a computerized scanning capillary viscometer (Hemathix®, Health Onvector, NJ) immediately after the carotid ultrasonography. The scanning capillary viscometer computes fluid viscosity using the Casson equation [17], which provides a relationship between the shear stress (τ) and shear rate ($\dot{\gamma }$) [18,19]. The wall shear stress and shear rate in the Casson equation were calculated from flow velocity and pressure drop measurements in a U-shaped capillary tube. Qualitatively, the fluid dynamic theory used in the scanning capillary viscometer is similar to the Poiseuille flow relation, which describes BV (*μ*) as:

$$\mu =\frac{\pi d^{4}△P}{128\mathrm{QL}}$$

where *Q* is the volume flow rate of blood, *d* is the inside diameter of a capillary tube, △P is the pressure drop along the capillary tube length, *μ* is BV, and *L* is the length of the tube.

#### *Shear rate* ($\dot{\gamma }$) *specific blood viscosity*

Shear rate specific BV values were determined at the corresponding PS/ED shear rates (i.e., *μ*_PS_ and *μ*_ED_) in each carotid arterial segment using the two Casson model constants (*k* and τ_y_):

$$\mathrm{Blood}\;\mathrm{vis}\text{cos}\mathrm{ity}\left(\mu \right)=\frac{\tau }{\dot{\gamma }}=\frac{\tau_{y}}{\dot{\gamma }}+k+2\sqrt{\frac{k\cdot \tau_{y}}{\dot{\gamma }}}$$

#### Blood viscosity at shear rate of 300 s^-1^ and of hematocrit-derived

Hematocrit-derived BV (*μ*_Hct_) was calculated using the formula [20]:

$$\begin{array}{c} \mathrm{BV}\;\left(\mu_{\mathrm{Hct}},\;\mathrm{centiPoise},\;\mathrm{cP}\right)\\ \;=1.4175+5.878H-12.98H^{2}+31.964H^{3} \end{array}$$

where H meant hematocrit (%)/100.

Values of BV at 300 s^-1^ (*μ*_300 s-1_) were chosen from the measurements using the computerized scanning capillary viscometer.

### Shear stress

The wall shear stresses specific to PS/ED BV values (e.g., *μ*_300 s-1_, *μ*_Hct_, and *μ*_PS_ and *μ*_ED_) and PS/ED shear rates were calculated as follows:

$$\begin{array}{l} \mathrm{PS}\;\mathrm{shear}\;\mathrm{stress}\;\left(\tau_{\mathrm{PS}}\right)={\dot{\gamma }}_{\mathrm{PS}}\;\times \;\left(\mu_{\mathrm{PS}}\;\mathrm{or}\;\mu_{300\;\text{s}-1}\;\mathrm{or}\;\mu_{\text{Hct}}\right)\\ \mathrm{ED}\;\mathrm{shear}\;\mathrm{stress}\;\left(\tau_{\mathrm{ED}}\right)={\dot{\gamma }}_{\mathrm{ED}}\;\times \;\left(\mu_{\mathrm{ED}}\;\mathrm{or}\;\mu_{300\;\text{s}-1}\;\mathrm{or}\;\mu_{\text{Hct}}\right) \end{array}$$

### Statistical analysis

Descriptive data for the major characteristics were expressed as means ± standard deviations (SD) or percentage as appropriate. An independent t-test was used to determine the statistical differences in the continuous variables, whereas a chi-square test was used for categorical variables. For comparison of adjusted PS/ED shear stresses, analysis of covariance was performed separately in the carotid segments and expressed as adjusted means ± standard errors. The mean values of PS/ED shear stresses using 3 methods of BV determination were compared with paired t-test. All statistical analyses were conducted using PASW Statistics 18 (SPSS, Chicago, IL).

## Results

Seventeen patients with lacunar infarction and 20 control subjects were enrolled, as shown in Table 1. All the demographic characteristics and major CVD risk factors were not different between cases and controls. Triglyceride and HDL cholesterol showed differences of borderline significance. Plasma tHcy, hematocrit, and blood viscosities of both calculated and measured were not significantly different.

**Table 1:** Characteristics of subjects

	**Control**	**Lacunar infarction**	p^*^
	**(n = 20)**	**(n = 17)**	
Age, y	64.7 ± 8.0	68.3 ± 7.7	0.181
Women,%	40.0	35.3	0.769
Smoker, ex- and current,%	25.0	41.2	0.295
Hypertension,%	65.0	64.7	0.985
Type 2 diabetes mellitus,%	35.0	23.5	0.447
Hypercholesterolemia,%	45.0	23.5	0.173
Total cholesterol, mmol/L	4.8 ± 1.1	4.6 ± 0.9	0.620
HDL cholesterol, mmol/L	1.2 ± 0.3	1.0 ± 0.3	0.095
Triglyceride, mmol/L	1.3 ± 0.6	2.4 ± 3.0	0.063
Plasma total Hcy, μmol/L	11.2 ± 4.0	12.3 ± 4.4	0.481
Hematocrit,%	42.0 ± 5.0	41.7 ± 6.5	0.892
Hematocrit-derived BV, cP	4.0 ± 0.6	4.0 ± 0.8	0.953
BV, cP, 5 s^-1^	11.2 ± 2.4	11.4 ± 4.2	0.840
100 s^-1^	4.7 ± 0.8	5.0 ± 1.3	0.451
300 s^-1^	4.0 ± 0.7	4.3 ± 1.2	0.364

Means ± standard deviations (SD) or percentages were expressed. HDL; high-density lipoprotein, Hcy; homocysteine, BV; blood viscosity.

Carotid IMT showed significantly higher values in patients with lacunar infarction than the control subjects only at the right bulb region, as shown in Table 2. The left CCA also showed higher IMT values in the patients, which reached significance. On the contrary, all the PS and ED shear rates at the both-sided CCAs were significantly lower in the patients with lacunar infarction than the controls. The PS and ED shear stresses at the both CCAs using shear rate specific BVs were significantly lower in the patients with lacunar infarction than the controls, even adjusted for age, sex, and cardiovascular risk factors, as shown in Table 3. Shear stresses of the both CCAs using blood viscosities at 300 s^-1^ or hematocrit-derived BV also showed significant differences between the two groups. There was no significant difference of the PS and ED shear stresses at the both CCAs according to the laterality of lacunar infarction (data not shown).

**Table 2:** Carotid intima-media thickness along carotid arteries, and peak-systolic (PS) and end-diastolic (ED) shear rates in both common carotid arteries

	**Control**	**Lacunar infarction**	p^*^
	**(n = 20)**	**(n = 17)**	
Intima-media thickness, cm			
Right ICA	0.9 ± 0.3	0.8 ± 0.2	0.243
Bulb	1.0 ± 0.2	1.2 ± 0.2	0.039
CCA	1.0 ± 0.3	1.1 ± 0.3	0.650
Left ICA	0.9 ± 0.2	0.8 ± 0.2	0.250
Bulb	1.1 ± 0.3	1.2 ± 0.2	0.141
CCA	1.0 ± 0.2	1.2 ± 0.3	0.051
Shear rate, s^-1^			
Right CCA, PS	405.7 ± 211.5	288.4 ± 68.5	0.028
ED	142.9 ± 98.8	97.0 ± 32.7	0.062
Left CCA, PS	503.8 ± 192.4	330.8 ± 109.9	0.002
ED	201.2 ± 117.2	120.2 ± 55.9	0.013

Means ± SDs were expressed.

*ICA*; internal carotid artery, *CCA*; common carotid artery.

**Table 3:** Adjusted^* ^**(mean ± SE) peak-systolic and end-diastolic shear stresses (dyne/cm**^**2**^**) of both common carotid arteries**

	**Control**	**Lacunar infarction**	***p***
	**(n = 20)**	**(n = 17)**	
Shear stress using shear-rate specific BV			
Right CCA, PS	16.0 ± 1.0	12.2 ± 1.1	0.028
ED	6.2 ± 0.4	4.7 ± 0.5	0.044
Left CCA, PS	19.5 ± 1.3	13.0 ± 1.3	0.005
ED	9.1 ± 0.9	5.0 ± 1.0	0.010
Shear stress using BV at 300 s^-1^			
Right CCA, PS	16.5 ± 1.2	12.0 ± 1.2	0.026
ED	5.7 ± 0.5	3.9 ± 0.5	0.041
Left CCA, PS	21.0 ± 1.5	12.7 ± 1.7	0.003
ED	8.9 ± 1.0	4.1 ± 1.0	0.008
Shear stress using Hct-derived BV			
Right CCA, PS	16.6 ± 1.3	11.0 ± 1.4	0.013
ED	5.8 ± 0.6	3.6 ± 0.6	0.033
Left CCA, PS	20.8 ± 1.6	11.9 ± 1.7	0.003
ED	9.1 ± 1.2	3.8 ± 1.2	0.012

^*^ Adjusted for age, sex, smoking, hypertension, type 2 diabetes, hypercholesterolemia, hematocrit, plasma tHcy, total cholesterol, triglyceride, and HDL cholesterol.

PS and ED shear stresses of CCAs and blood viscosities (shear rate specific BV, BV at 300 s^-1^, and Hct-derived BV) were measured, as shown in Table 4. Compared with the shear stresses using shear rate specific BV, overall 4.5% higher and 7.3% lower PS and ED shear stresses were calculated using the two other BVs (Hct-derived BV or BV at 300 s^-1^). Blood viscosities showed 0.9% and 13.0% differences for PS and ED phases, respectively. There was no significant difference between BV of hematocrit-derived and BV at 300 s^-1^, and also for their calculated shear stresses.

**Table 4:** **Comparisons of mean values (± SD, n=37) of PS and ED shear stresses and BVs according to applications of shear rate specific BV (A), BV at 300 s**^**-1 **^**(B), and hematocrit-derived BV (C)**

	**Shear rate-specific BV (A)**	**BV at shear rate 300 s**^**-1 **^**(B)**	**Hematocrit-derived BV (C)**	p^*^		
				**A vs. B**	**A vs. C**	**B vs. C**
Shear stress, dyne/cm^2^						
Right CCA						
PS	14.0 ± 6.4	14.4 ± 7.2	14.4 ± 8.2	0.047	0.605	0.753
ED	5.5 ± 3.0	4.9 ± 3.1	5.0 ± 3.6	<0.001	0.010	0.935
Left CCA						
PS	16.2 ± 5.9	17.1 ± 6.8	17.1 ± 8.0	0.003	0.244	0.695
ED	7.0 ± 3.7	6.6 ± 4.1	6.7 ± 4.9	0.001	0.131	0.888
Blood viscosity, cP						
Right CCA						
PS	4.1 ± 1.0	4.2 ± 0.9	4.0 ± 0.7	0.268	0.364	0.153
ED	4.8 ± 1.2	4.2 ± 0.9	4.0 ± 0.7	<0.001	<0.001	0. 153
Left CCA						
PS	4.0 ± 1.0	4.2 ± 0.9	4.0 ± 0.7	0.003	0.980	0. 153
ED	4.6 ± 1.2	4.2 ± 0.9	4.0 ± 0.7	<0.001	<0.001	0. 153

## Discussion

The present study introduces a novel method for calculating site-specific vascular shear at the CCA using dynamic measures of shear rate and BV. For the calculation, Casson model constants (*k* and *τ*_*y*_) were derived from BV data obtained using a computerized scanning capillary viscometer. In this study, we demonstrate that shear stress is a dynamic parameter that varies widely along the carotid artery. With the novel method, vascular shear stress at the both CCAs was significantly lower in patients with lacunar infarction than the control subjects. Although CVD risk factors were not significantly different between the two groups, and even carotid IMT showed variable findings, vascular shear stresses at the CCAs showed discriminative features. Low shear stress along carotid artery was reported to be associated with ischemic stroke of large artery atherosclerosis, especially in the same side to the affected hemisphere [21]. To our knowledge, this study is the first to compare vascular shear stress in the patients with lacunar infarction with control subjects, on a shear rate specific basis using the individual subject’s own BV profile.

The vascular shear stress of the CCAs, irrespective of the methods of shear stress calculation, showed similar patterns of difference between the patients with lacunar infarction and controls, as shown in Table 3. But, there were significant differences for the mean values of CCA shear stresses and blood viscosities according to the application methods of BV. Using the shear rate specific BV, the lowest values of BV were observed at the PS phase and the highest values were detected at the ED phase. As such, if blood is assumed to behave as a Newtonian fluid and a certain representative value is used, vascular shear stresses will be calculated as higher than their true values at the PS phases, and lower in the ED phases. In the present study, when shear rate specific BVs were used, PS and ED shear stresses were 4.5% higher and 7.3% lower than shear stresses using constant values of BV, as shown in Table 4. This discrepancy would become more pronounced in arterial segments with elevated pulsatility indexes [22]. While the diagnostic methods described herein require further validation, this rationale underscores the non-Newtonian behavior of blood flow and makes a meritorious case for further research.

Our study results are consistent with the report that lacunar infarction is associated with endothelial dysfunction [11], which is controlled by vascular shear stress [2]. Previously, we reported patients with lacunar infarction had lower blood flow velocities in cerebral arteries than patients with other ischemic stroke subtypes [12]. The both low vascular shear stress in the CCAs and low blood flow velocities in cerebral arteries of patients with lacunar infarction may foster understanding of the pathophysiology of the disease.

Low carotid arterial shear stress in the patients with lacunar infarction who have no obvious carotid stenosis suggests increased resistance in small cerebral arteries [23] that may potentially increase arterial pressure in the more proximate carotid arteries. The transmission of increased pressure drop of cerebral arteries would cause an increase of carotid arterial pressure, a reduction of flow velocity, a compensatory increase in arterial diameter, and a decrease of vascular shear stress. When the increase of arterial pressure and the decrease of shear stress reinforce themselves through a feedback loop toward greater instability, a vicious cycle might cause lacunar infarction. Although thrombosis due to endothelial denudation under high shear could occur [24,25], thrombosis in the milieu of low blood flow velocities and low shear stress may also increase the propensity for ischemic events, especially at vulnerable regions [26]. Under low shear, increased transudation of blood or its components in small arteries of the deep cerebral tissue might also occur through the increased porosity of gap junctions, the morphological changes of endothelial cells [27], to cause lacunar (small-sized) hemorrhage or potentially lacunar infarction [28].

The present study has several limitations. First, we used a local centerline velocity to determine a local shear rate, which is expressed as 4 V/D. This equation is derived from a parabolic velocity profile for a fully developed laminar flow in a straight circular tube [29]; so, the assumptions in this model have limitations for estimation of shear stress in regions of complex flow even in the relatively straight CCAs. However, the use of the centerline velocity together with 4 V/D expression allows the estimation of local changes in the velocity gradient if there is no significant hemodynamic compromise [30]. Further investigations are required to determine if centerline arterial shear stress is a parameter that has some critical threshold values for vascular diseases or pathologic conditions. Our study showed 76 carotid arteries in patients with lacunar infarction at CCA peak systolic shear stress of 12~13 dyne/cm^2^, which is slightly lower than the previous report of carotid arterial shear stress in the subjects with high risk vascular profiles [30] or with carotid plaque [31]. Second, there were differences of shear rate and shear stress between the right and left CCAs. That might be largely due to the differences of cerebral arterial resistance, and in part, the proximal arterial geometry: the right CCA comes from a celiac trunk and might have more tortuosity than the left CCA which comes from aorta directly. Third, the present cross-sectional study could not address the causal relationship between low carotid artery shear stress and lacunar infarction.

## Conclusion

We describe a new method to calculate dynamic vascular shear stress in human carotid arteries using flow rates obtained from ultrasonography and shear rate-specific BV. With the innovative method, the carotid arterial shear stress was significantly lower in patients with lacunar infarction than the control subjects. The prognostic contribution of decreased vascular shear stress to incident lacunar infarction requires further study.

## Competing interest

The authors declare that they have no competing interest.

## Authors’ contributions

SKJ participated in data collection, conceived the study, drafted and reviewed the manuscript. RSR conceived the present study and reviewed the manuscript. All authors read and approved the final manuscript.

## Pre-publication history

The pre-publication history for this paper can be accessed here:

http://www.biomedcentral.com/1471-2377/13/36/prepub
